# Temporal Dependence of the Phylogenetic Diversity–Habitat Size Relationship: Evidence from a Closed Fermentation System

**DOI:** 10.3390/microorganisms14071539

**Published:** 2026-07-14

**Authors:** Yiting Cheng, Wei Deng, Kun Tan, Wen Xiao

**Affiliations:** 1 Institute of Eastern-Himalaya Biodiversity Research, Dali University, Dali 671003, China; chengyt@eastern-himalaya.cn (Y.C.); dengw@eastern-himalaya.cn (W.D.); 2 Center for Interdisciplinary Sciences, Dali University, Dali 671003, China; 3 Provincial Innovation Team for Biodiversity Conservation and Utilization in the Three Parallel Rivers Region, Dali University, Dali 671003, China; 4 Collaborative Innovation Center for Biodiversity and Conservation in the Three Parallel Rivers Region of China, Dali 671003, China

**Keywords:** phylogenetic diversity–habitat size relationship, species–area relationship, temporal dynamics, community succession, microbial microcosm

## Abstract

Phylogenetic diversity–habitat size relationship (PDSR), as an extension of the species–area relationship, incorporates evolutionary history to further elucidate the mechanisms shaping biogeographic patterns. However, whether PDSR is temporally unstable during community succession, and the mechanisms underlying such instability, remain poorly understood. In this study, we used a fermentation microbial community as a model and established a closed microcosm system spanning a 10–1000 mL volume gradient. By continuous sampling over 0–60 days and 16S rRNA high-throughput sequencing, we characterized the dynamic changes in PDSR throughout succession. The results showed that PDSR underwent stage-specific shifts during community succession: it was significantly positive in the early stage, weakened or became non-significant in the middle stage, and turned negative or remained non-significant in the late stage. The early positive PDSR was mainly attributable to the greater retention of rare lineages in larger-volume habitats, whereas in the late stage, phylogenetic diversity increased in smaller-volume habitats, suggesting a positive role in the resuscitation of potentially dormant lineages. Phylogenetic beta-diversity analysis further showed that lineage turnover rate increased over time, with significant divergence among samples in the later stage. In addition, pH decreased significantly over succession and, through interactions with time and volume, exerted a marked influence on community phylogenetic structure. This study reveals the temporal dynamism of biodiversity spatial patterns and confirms that PDSR shifts non-steadily across successional stages, providing new insights into the organization of microbial diversity across multiple scales.

## 1. Introduction

How biodiversity changes across spatial scales is a central question in ecology. The species–area relationship (SAR) describes the common increase in species richness with sampling area or habitat size, and it has served as a key theoretical foundation for island biogeography, community ecology, and extinction-risk prediction [1,2,3]. In microbial ecology, bacterial communities can also exhibit taxon–area relationships and spatial scaling effects, indicating that scale dependence is not restricted to macroorganisms [4,5,6]. Nevertheless, SAR focuses on the number of species or taxa and implicitly treats all taxa as equivalent contributors to diversity. It therefore cannot distinguish whether increasing habitat size preserves closely related taxa or lineages representing longer evolutionary histories. Phylogenetic diversity (PD) integrates branch lengths into a phylogenetic tree and quantifies the total evolutionary history retained by a community [7,8]. The phylogenetic diversity–area relationship (PDAR) can thus be considered a phylogenetic extension of SAR and can be used to test whether spatial scaling also alters the retention of evolutionary history [9]. Recent studies on multifaceted diversity–area relationships have further shown that richness, phylogenetic diversity, and functional diversity may respond inconsistently to habitat size [10,11].

Biological communities undergo continuous succession, and their composition is shaped jointly by colonization, local extinction, species interactions, environmental filtering, dispersal, and stochastic processes. As community structure changes and modifies the local environment, the relative contributions of environmental filtering, competitive exclusion, dispersal, and stochasticity to community assembly may also shift [12,13,14,15]. Therefore, species diversity, phylogenetic structure, and functional diversity can all change over time, and the integration of phylogenetic biology with community ecology provides complementary information for inferring assembly processes [16]. Previous microbial microcosm studies have shown that SAR is not necessarily maintained throughout succession; instead, it can appear, weaken, or disappear at different stages [17]. Although PDAR and SAR may exhibit similar volume responses in some cases, they can also be decoupled, with richness and phylogenetic diversity responding differently to habitat size. Such decoupling may be related to the depth of lineage turnover, phylogenetic tree structure, the organization of the regional species pool, and local filtering processes [9,18]. It is therefore necessary to test whether PDAR varies temporally during community succession and whether its trajectory remains consistent with SAR.

Microbial microcosm systems are characterized by short generation times, rapid community reassembly, and high experimental controllability, making them an ideal model for investigating the temporal dynamics of diversity–volume relationships. In this study, we used the microbial community of paocai fermentation as a model system and established closed liquid microcosms spanning a volume gradient of 10–1000 mL to represent available habitat size. High-temporal-resolution data on community succession were obtained throughout 1–60 d of fermentation. Unlike previous SAR and PDAR studies that have mainly focused on spatial patterns, this study shifted the measurement scale from conventional ‘area’ to ‘volume’. This adjustment is motivated by the three-dimensional nature of microbial habitats. In closed liquid microcosms, microorganisms are suspended within a three-dimensional aqueous environment, and their available space, total initial resources, population carrying capacity, and accumulation of metabolic products are all directly governed by liquid volume rather than by two-dimensional projected area. Therefore, in this experimental system, volume better captures the habitat boundaries, resource conditions, and environmental constraints actually experienced by microorganisms than area does. Here, we define the relationship between community phylogenetic diversity and habitat size as the phylogenetic diversity–habitat size relationship (PDSR) and examine whether community PD changes systematically with available habitat size.

## 2. Materials and Methods

### 2.1. Construction of Microbial Microcosms

We constructed closed paocai fermentation microcosms and used sterile glass bottles of different volumes to simulate isolated liquid habitat patches of different sizes. In this study, habitat size was represented by liquid volume rather than two-dimensional area because a closed liquid microcosm does not have an obvious planar area equivalent to that in natural landscapes. Using volume to represent the size of microbial “islands” has a precedent in microcosm studies [5].

All starter brine was prepared in a single batch. Briefly, 35 kg cabbage, 35 kg white radish, 1 kg ginger, 1 kg Sichuan pepper, 2 kg chili pepper, 2.5 kg rock sugar, and 210 kg purified water containing 6% salt were mixed. All raw materials were purchased from local markets and kept fresh and clean before the experiment. After thorough mixing, the materials were distributed into fermentation jars, sealed, and fermented for 7 d at room temperature (approximately 18–22 °C). After fermentation, solids were removed through sterile gauze, and the filtrate was collected as the paocai starter brine. Approximately 200 kg of starter brine was obtained. Before dispensing, the brine was mixed thoroughly again, and all microcosms received the same well-mixed source brine to minimize initial differences in resources, energy supply, and microbial community composition. The brine was then distributed into sterile glass bottles according to the volume treatments. All bottles were sealed with caps and sealing film and incubated at 25 °C.

Before the experiment, three initial samples were collected from the well-mixed starter brine to characterize the initial community. Seven habitat-size treatments were established: 10, 20, 50, 100, 250, 500, and 1000 mL, with three replicates for each volume. Continuous time-series monitoring was performed from 1 to 60 d. The sampling frequency was set according to the expected rate of community succession: samples were collected daily on days 1–10, every 2 d on days 11–30, and every 5–6 d on days 31–60. In total, 26 microcosm sampling time points were obtained: 1, 2, 3, 4, 5, 6, 7, 8, 9, 10, 12, 14, 16, 18, 20, 22, 24, 26, 28, 30, 35, 40, 45, 50, 55, and 60 d. Including the initial starter-brine samples, the experiment contained 549 samples.

### 2.2. Sampling, DNA Extraction, 16S rRNA Amplicon Sequencing, and Sequence Processing

Before sampling, the brine in each bottle was mixed thoroughly to reduce the influence of within-bottle spatial heterogeneity. An appropriate volume of brine was then collected for DNA extraction. Samples were immediately centrifuged at 8000 r min^−1^ for 10 min, and the pellet was used for microbial DNA extraction and stored at −80 °C. All samples were transported on dry ice to the sequencing company for amplicon sequencing.

The experiment adopted a destructive sampling design. For each sampling time point and volume treatment, replicate samples corresponded to independent microcosms; each bottle was sampled only once at its designated time point. This design avoided repeated sampling-induced changes in intrabottle resources, microbial density, headspace gas ratio, and metabolic-product accumulation, thereby reducing potential sampling disturbances to the community succession process. DNA extraction, PCR amplification, library preparation, and sequencing were conducted using a unified workflow. During downstream processing, samples from any single time point or volume treatment were, as far as possible, not processed as an isolated batch, in order to reduce the potential influence of technical batch effects on estimates of volume effects.

Total genomic DNA was extracted from all samples using the cetyltrimethylammonium bromide (CTAB) method. DNA concentration and integrity were checked using 2% agarose gel electrophoresis, and the extracted DNA was used as the PCR template. The V3–V4 region of the bacterial 16S rRNA gene was amplified using primers 338F (5′-ACTCCTACGGGAGGCAGCAG-3′) and 806R (5′-GGACTACHVGGGTWTCTAAT-3′). PCR amplification was performed using a T100 Thermal Cycler (Bio-Rad Laboratories, Inc., Hercules, CA, USA). The PCR protocol consisted of initial denaturation at 95 °C for 3 min; 27 cycles of 95 °C for 30 s, 55 °C for 30 s, and 72 °C for 45 s; and final extension at 72 °C for 10 min.

PCR products were checked by 2% agarose gel electrophoresis, and target bands were excised and purified using the AxyPrep DNA Gel Extraction Kit (Axygen, Inc., Union City, CA, USA). Purified PCR products were quantified using a QuantiFluor™-ST fluorometer (Promega Corporation, Madison, WI, USA). Amplicons from all samples were pooled in equimolar amounts and sequenced on an Illumina NovaSeq platform (Illumina, Inc., San Diego, CA, USA) using paired-end sequencing.

Raw sequencing data were processed using QIIME 2, release 2019.1 [19]. First, FASTQ data were imported into QIIME 2, and sequences from each sample were quality filtered, trimmed, denoised, merged, and checked for chimeras. Amplicon sequence variants (ASVs) were inferred using the q2-dada2 plugin implemented in QIIME 2 [20]. Representative ASV sequences were taxonomically assigned using the Greengenes 13_8 99% database [21]. After taxonomic annotation, mitochondrial, chloroplast, and other non-target sequences were removed. The resulting ASV feature table, taxonomy table, and representative sequences were used for subsequent analyses of community composition and phylogenetic structure.

### 2.3. Data Integration, Phylogenetic Tree Construction, and Definition of Analytical Objects

The ASV feature table, taxonomy table, representative sequences, phylogenetic tree, sample metadata, and environmental factor table were integrated in R. Sample names and ASV identifiers were first standardized, and only samples and ASVs shared among the ASV table, taxonomy table, phylogenetic tree, and metadata were retained. Volume was recorded in mL and log10 transformed in models evaluating habitat-size effects. Time was recorded as culture day. Community developmental stages were defined according to sampling time as early (1–10 d), middle (12–30 d), and late (35–60 d) stages. This classification took into account the sampling frequency, pH trajectories, and fermentation succession process: the early stage corresponded to rapid community establishment and a rapid decline in pH; the middle stage represented a transition period during which pH approached a relatively low level and dominant taxa were replaced; and the late stage corresponded to a relatively mature, acidified fermentation phase with intensified resource limitation.

The phylogenetic tree was constructed from representative ASV sequences within the QIIME 2 workflow. First, representative ASV sequences were aligned using MAFFT [22]. Ambiguously aligned sites or sites with limited phylogenetic information were then removed using a masking step, retaining more conserved alignment regions for downstream inference (parameters: mask_max_gap_frequency = 1.0 and mask_min_conservation = 0.4). To balance phylogenetic accuracy with computational efficiency, the masked alignment was used to infer an unrooted phylogenetic tree with FastTree under the nucleotide model and default settings [23]. The resulting tree was subsequently midpoint-rooted. Branch lengths were estimated during FastTree inference and retained in the rooted tree as evolutionary distances among representative ASV sequences. All phylogenetic diversity and UniFrac distance analyses were conducted using the same rooted phylogenetic tree matched to the ASV feature table [24,25]. Before analysis, the tree tip names were checked against the ASV table row names, and only shared ASVs were retained. The final integrated ASV abundance matrix, sample metadata, taxonomic annotation table, and phylogenetic tree were used to construct a phyloseq object, which served as the basis for subsequent analyses of diversity, community composition, turnover, and assembly processes. Construction and integration of the phyloseq object followed the standard data structure of the phyloseq package [26].

### 2.4. Calculation of ASV Diversity and Phylogenetic Diversity

To evaluate temporal changes in community diversity among microcosms of different volumes, we calculated ASV richness, Shannon diversity, Simpson diversity, Pielou evenness, and total sequence abundance. ASV richness was calculated as the number of non-zero ASVs in each sample, while Shannon and Simpson indices were calculated from the ASV abundance matrix.

Phylogenetic diversity metrics included Faith’s PD, mean pairwise distance (MPD), and mean nearest taxon distance (MNTD). Faith’s PD represents the total branch length covered by all ASVs in a sample; MPD represents the mean phylogenetic distance among all pairs of ASVs in a community and reflects deeper phylogenetic structure; and MNTD represents the mean distance from each ASV to its nearest relative and reflects shallower phylogenetic structure. Together, these metrics characterize phylogenetic structure in terms of total branch length, overall relatedness, and nearest-neighbor relatedness [7,8,27].

### 2.5. Daily Habitat-Size Effects and Their Temporal Dependence

To test whether the effects of habitat size on ASV diversity and phylogenetic diversity changed over time, we fitted a linear model at each sampling day in the form diversity metric ~log10(Volume). Metrics analyzed included ASV richness, Faith’s PD, MPD, and MNTD. The model slope represented the direction and strength of the habitat-size effect at each time point: a positive slope indicated higher diversity or phylogenetic distance in larger-volume habitats, whereas a negative slope indicated lower diversity or phylogenetic distance in larger-volume habitats.

To reduce the risk of false positives caused by daily multiple testing, the P-values from the 26 daily regressions for each metric were adjusted using the Benjamini–Hochberg false discovery rate (FDR) method [28]. In addition to daily models, we fitted an overall interaction model in the form metric_z ~ Days_z × log10(Volume)_z to test whether the habitat-size effect changed systematically over time. In the environmental-factor analyses, we compared a baseline model, a model including pH, and a model including pH interaction terms.

For each sampling day, the regression slope, standard error, R^2^, and P-value were extracted, and slope significance was evaluated after FDR correction. To visualize temporal changes in habitat-size effects, daily slopes and standard errors were plotted as time-series bar charts. We also calculated the mean and standard error of each diversity metric for each volume and time point and plotted temporal trajectories for each volume treatment.

### 2.6. ASV Abundance-Class Structure and Major ASV Composition

To clarify the compositional basis of diversity changes, ASVs in each sample were classified into abundance classes according to their relative abundance: dominant ASVs (>1%), intermediate ASVs (0.01–1%), and rare ASVs (<0.01%). The number of ASVs in each abundance class was then calculated for each sample [29,30,31,32]. Mean values for each abundance class were summarized by sampling day and volume treatment to show changes in ASV abundance-class structure across time and habitat size. Samples with zero sequencing results were excluded from relative-abundance and abundance-class calculations and were shown as blank bars in the corresponding figures.

To analyze the composition of major ASVs, relative ASV abundances were calculated for each sample, and ASVs with relatively high mean abundance or high occurrence frequency across samples were selected as major ASVs. Major ASVs were displayed separately, whereas the remaining low-abundance or non-major ASVs were merged as “Others”. Mean relative abundances of major ASVs were summarized by time point and volume treatment and visualized as stacked bar plots to compare shifts in dominant community composition over time and among volumes.

### 2.7. Explanatory Role of pH in Diversity and Phylogenetic Structure

To evaluate whether environmental change contributed to temporal dynamics in diversity and phylogenetic structure, pH data were merged with the diversity table by sample name. First, pH trajectories were plotted for each volume treatment, and the model pH_z ~ Days_z × log10(Volume)_z was fitted to test whether pH changed with time, habitat size, and their interaction. All continuous explanatory variables were standardized in multivariate models.

We then analyzed relationships between pH and ASV richness, Faith’s PD, MPD, and MNTD. Pearson and Spearman correlations were calculated, and scatterplots with regression lines were generated for pH and each diversity or phylogenetic metric. To compare the explanatory contribution of pH, three types of linear models were fitted: the baseline model metric_z ~ Days_z × log10(Volume)_z; the pH-additive model metric_z ~ Days_z × log10(Volume)_z + pH_z; and the interaction model metric_z ~ Days_z × log10(Volume)_z + pH_z + Days_z:pH_z + log10(Volume)_z:pH_z. Changes in adjusted R^2^ after adding pH were used to evaluate the incremental explanatory contribution of pH.

In addition, for each sampling day, daily models with and without pH control were fitted, and the slopes of log10(Volume) before and after controlling for pH were compared. This analysis was used to determine whether pH could explain, or partially explain, the temporal dependence of habitat-size effects.

### 2.8. ASV Turnover and Bray–Curtis Beta-Diversity Decomposition

To identify the sources of compositional change between adjacent time intervals, ASV composition was compared between adjacent sampling days within each volume treatment. Based on ASV presence–absence information, ASV persistence, ASV loss, ASV gain, delta richness, and turnover rate were calculated. ASV loss represented the number of ASVs present at the previous time point but not detected at the subsequent time point; delta richness represented the difference in ASV richness between two adjacent time points; and turnover rate represented the relative intensity of ASV replacement between adjacent time points.

In parallel, Bray–Curtis beta diversity between adjacent time intervals was decomposed into similarity, turnover, and nestedness components [33,34], allowing the distinction between community differences caused mainly by compositional replacement and those caused by nested loss. To test whether turnover metrics were affected by habitat size, the model turnover metric ~ log10(Volume) was fitted separately for each adjacent time interval, and slopes and *p*-values were extracted.

### 2.9. Abundance-Weighted Phylogenetic Beta-Diversity Analysis

To evaluate differences in dominant lineage composition among fermentation stages and volume treatments, replicate samples from the same sampling day and volume treatment were merged into one assemblage, and only assemblages containing at least five ASVs were retained. After matching the ASV abundance matrix with the phylogenetic tree, ASV abundances were converted into an assemblage-by-phylogenetic-branch abundance matrix according to the tree and weighted by branch length. Branch abundances in each assemblage were then relativized and Hellinger transformed, and abundance-weighted phylogenetic Hellinger distance was calculated using Euclidean distance [35].

Principal coordinates analysis (PCoA) was performed based on the resulting distance matrix to visualize changes in phylogenetic community composition across time, stage, and volume treatment. Samples were classified into Early (≤10 d), Middle (10–30 d), and Late (>30 d) stages. The betadisper procedure was used to test differences in within-group phylogenetic dispersion among stages and volume treatments, and PERMANOVA was used to test the effects of Day, log10(Volume), and their interaction on abundance-weighted phylogenetic beta diversity [36,37]. Pairwise phylogenetic distances among assemblages from different time points within the same volume treatment were also calculated to analyze changes in phylogenetic beta diversity across adjacent time intervals and across increasing time lags.

### 2.10. Statistical Analysis and Visualization

All statistical analyses and visualizations were performed in R version 4.6.0 (R Foundation for Statistical Computing, Vienna, Austria). ASV data organization, grouped summaries, and standardization were conducted using tidyverse version 2.0.0. Phylogenetic tree handling and Faith’s PD calculation were performed using ape version 5.8-1 and picante version 1.8.2 [38]. Community distance calculations, PCoA, PERMANOVA, and betadisper analyses were mainly performed using phyloseq version 1.56.0 and vegan version 2.7-5 [26,36,37]. Figures were produced and combined using ggplot2 version 4.0.3, patchwork version 1.3.2, and cowplot version 1.2.0. *p*-values from the daily regressions were corrected using the Benjamini–Hochberg false discovery rate (FDR) method [28]. The PERMANOVA and PDSR null-model analyses were based on 999 permutations or randomizations. Unless otherwise stated, the significance threshold was *p* < 0.05.

## 3. Results

### 3.1. Habitat-Size Effects on ASV Richness and Faith’s PD Shifted Across Successional Stages

**Figure 1:**
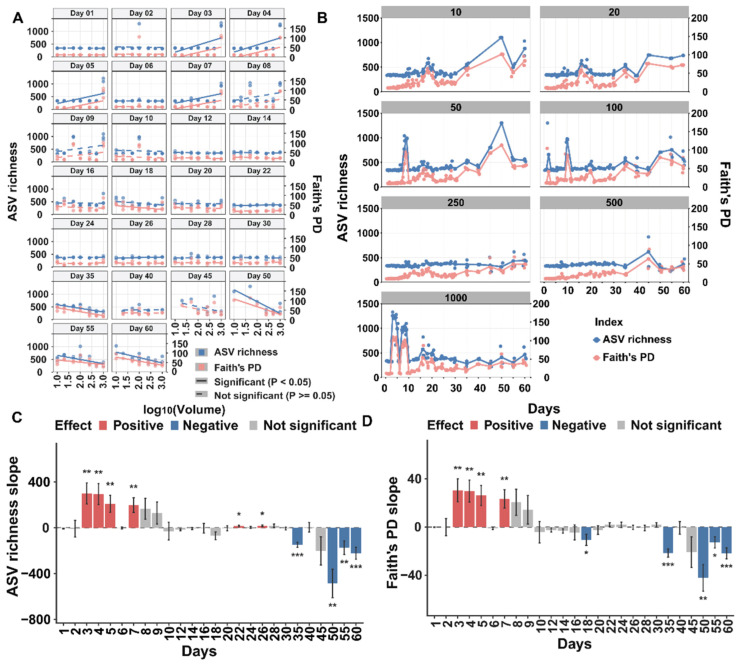
Temporal dynamics of ASV richness and Faith’s PD under different habitat sizes. (**A**) Daily relationships between ASV richness or Faith’s PD and log10(Volume). (**B**) Temporal trajectories of ASV richness and Faith’s PD under different volume treatments. (**C**) Temporal changes in the slope of the volume effect on ASV richness. (**D**) Temporal changes in the slope of the volume effect on Faith’s PD. Slopes were estimated from linear models fitted separately for each sampling day. Red bars indicate significant positive effects, blue bars indicate significant negative effects, and gray bars indicate non-significant effects. Error bars indicate standard errors, and asterisks indicate significance levels (* *p* < 0.05, ** *p* < 0.01, and *** *p* < 0.001).

ASV richness and Faith’s PD both exhibited clear temporal dynamics, and their relationships with habitat size changed in direction and strength during community development. Daily linear models showed that, in the early stage, both SAR and PDSR were positive, although effect strength gradually weakened. The slopes of ASV richness against log10(Volume) on days 3, 4, 5, and 7 were 300.58, 295.62, 210.34, and 199.05, respectively, and the corresponding slopes of Faith’s PD were 30.49, 29.78, 26.27, and 23.47, all of which were significant after FDR correction (Figure 1A,C,D). From days 10 to 28, positive volume effects on ASV richness and Faith’s PD weakened substantially. On day 18, a negative PDSR was detected while SAR remained significantly positive, indicating that the responses of the two metrics to the volume gradient had begun to diverge. In the third stage, SAR and PDSR were mostly negative or non-significant. On days 35, 50, 55, and 60, the slopes of ASV richness were −147.54, −484.71, −172.43, and −220.87, respectively, and the corresponding slopes of Faith’s PD were −21.62, −42.21, −12.76, and −21.85.

Temporal trajectories among volume treatments further supported this transition (Figure 1B). In the early stage, larger-volume treatments, especially 1000 mL, generally had higher ASV richness and Faith’s PD. In the middle stage, differences among volume treatments narrowed and diversity levels converged. In the late stage, ASV richness and Faith’s PD decreased markedly in some large-volume treatments, whereas small or intermediate volumes maintained higher diversity. Overall, the habitat-size effects on ASV richness and Faith’s PD were not stable positive relationships but underwent a stage-dependent transition from early positive effects to middle-stage weakening and divergence and, finally, to late negative or non-significant effects.

### 3.2. MPD and MNTD Revealed Different Responses of Deep and Shallow Phylogenetic Structure

**Figure 2:**
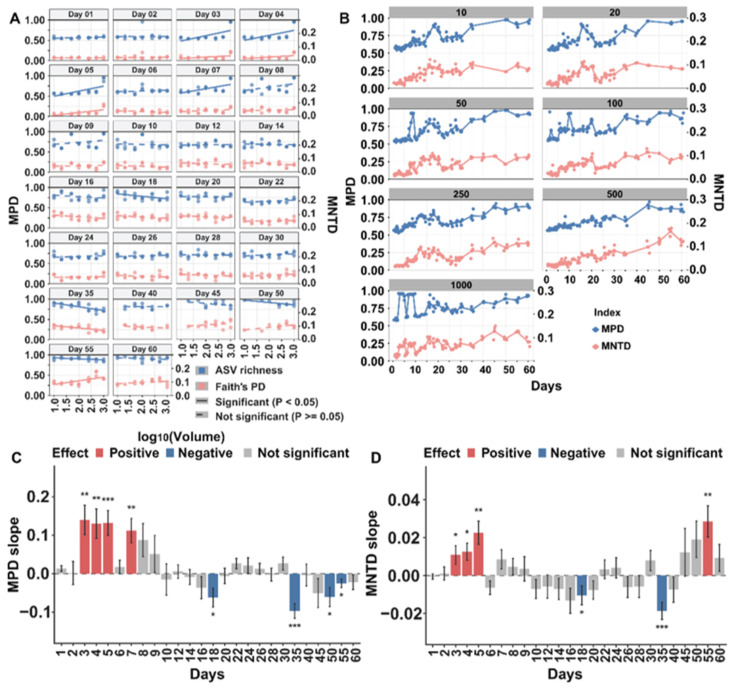
Temporal dynamics of MPD and MNTD under different habitat sizes. (**A**) Changes in habitat-size effect slopes for MPD and MNTD through time. (**B**) Temporal trajectories of MPD and MNTD under different volume treatments. (**C**) Temporal changes in the habitat-size effect slope for MPD. (**D**) Temporal changes in the habitat-size effect slope for MNTD. Slopes were estimated from linear models fitted separately at each sampling day, with phylogenetic structure metrics as responses and log10-transformed volume as the predictor. Red bars indicate significant positive effects, blue bars indicate significant negative effects, and gray bars indicate non-significant effects. Error bars indicate standard errors, and asterisks indicate significance levels (* *p* < 0.05, ** *p* < 0.01, and *** *p* < 0.001).

Daily slope analyses showed that volume had significant positive effects on MPD on days 3, 4, 5, and 7, but the effect became significantly negative on day 35. For MNTD, volume had a significant positive effect on day 5, a significant negative effect on day 35, and a significant positive effect again on day 55 after FDR correction (Figure 2A,C,D). Across volume treatments, both MPD and MNTD generally increased over fermentation time (Figure 2B). In the overall model for MPD, the adjusted R^2^ of the baseline model was 0.442, and Days_z, log10(Volume)_z, and their interaction were all significant (Days_z: β = 0.649, *p* = 5.68 × 10^−64^; log10(Volume)_z: β = 0.100, *p* = 0.003; interaction: β = −0.166, *p* = 1.05 × 10^−6^), indicating that MPD was jointly affected by succession time, habitat volume, and their interaction. For MNTD, the time effect was stronger, but neither the volume main effect nor the Days_z × log10(Volume)_z interaction was significant in the baseline model. The adjusted R^2^ of the MNTD baseline model was 0.540, with a significant positive Days_z effect (β = 0.730, *p* = 7.46 × 10^−87^), whereas log10(Volume)_z and the interaction had β = 0.028, *p* = 0.350 and β = 0.034, *p* = 0.267, respectively.

### 3.3. Rare ASV Retention and Dominant-Taxon Replacement Formed the Compositional Basis of PDSR Changes

**Figure 3:**
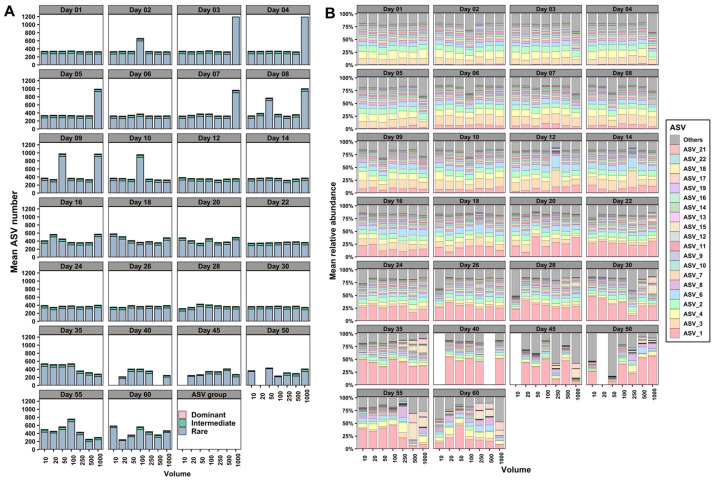
ASV abundance-class structure and major ASV relative-abundance composition across time and volume treatments. (**A**) Mean numbers of dominant, intermediate, and rare ASVs across time points and volumes. (**B**) Mean relative-abundance composition of major ASVs across time points and volumes. Taxonomic annotations of major ASVs were used to interpret shifts in dominant groups: fermentation-associated lactic acid bacteria mainly included *Lactobacillus*, *Lactococcus*, and *Leuconostoc*, whereas Proteobacteria-related taxa mainly included *Enterobacteriaceae* such as *Citrobacter* and *Pantoea*. “Others” represents all ASVs other than the selected major ASVs. Blank bars indicate volume-by-time combinations with zero sequencing results and therefore no available sequences for calculating abundance classes or relative composition.

ASV abundance-class analysis showed that total ASV numbers were dominated by rare ASVs (Figure 3A). Across all time points and volume treatments, the average numbers of dominant, intermediate, and rare ASVs were 16.06, 44.27, and 335.49, respectively. Among treatment combinations with non-zero total ASV numbers, rare ASVs accounted for more than 90% of total ASVs on average, indicating that rare taxa were the major source of ASV richness variation and early habitat-size effects.

Total ASV numbers varied greatly among volumes and time points, ranging from 217 to 1255.67 among non-zero combinations. The maximum value occurred on day 3 in the 1000 mL treatment, where the mean total ASV number was 1255.67 and the mean number of rare ASVs was 1192.67, accounting for 95.0% of total ASVs. This pattern was consistent with the early positive ASV richness–volume relationship and indicated that larger volumes increased total ASV richness mainly by retaining more rare ASVs during the initial community-establishment stage.

Taxonomic interpretation of the major ASVs shown in the stacked bar plots indicated that the 4714 ASVs mainly belonged to *Proteobacteria* (2318 ASVs, 49.2%) and *Firmicutes* (1243 ASVs, 26.4%), followed by Actinobacteria (397 ASVs, 8.4%) and Bacteroidetes (348 ASVs, 7.4%). Among fermentation-associated dominant groups, Lactobacillales included Lactobacillaceae, Leuconostocaceae, and Streptococcaceae, with *Lactobacillus*, *Lactococcus*, and *Leuconostoc* represented by 85, 11, and 15 ASVs, respectively. Enterobacteriales were mainly represented by Enterobacteriaceae, including genera such as *Citrobacter* and *Pantoea*.

Thus, changes in the major-ASV stacked plots can be interpreted as a shift in relative dominance between fermentation-associated lactic acid bacteria and Proteobacteria-related taxa. In the early stage, larger volumes retained more rare ASVs and more diverse lineages, leading to higher ASV richness and Faith’s PD. As acidification progressed, acid-tolerant or fermentation-associated taxa such as *Lactobacillus*, *Lactococcus*, and *Leuconostoc* gradually increased in relative dominance, while some Enterobacteriaceae and other low-abundance lineages were lost or replaced. This process weakened the early lineage-retention advantage conferred by larger volume and helps explain the weakening or reversal of Faith’s PD–habitat volume relationship in the middle and late stages.

### 3.4. Acidification Contributed to Temporal Changes in Phylogenetic Structure

**Figure 4:**
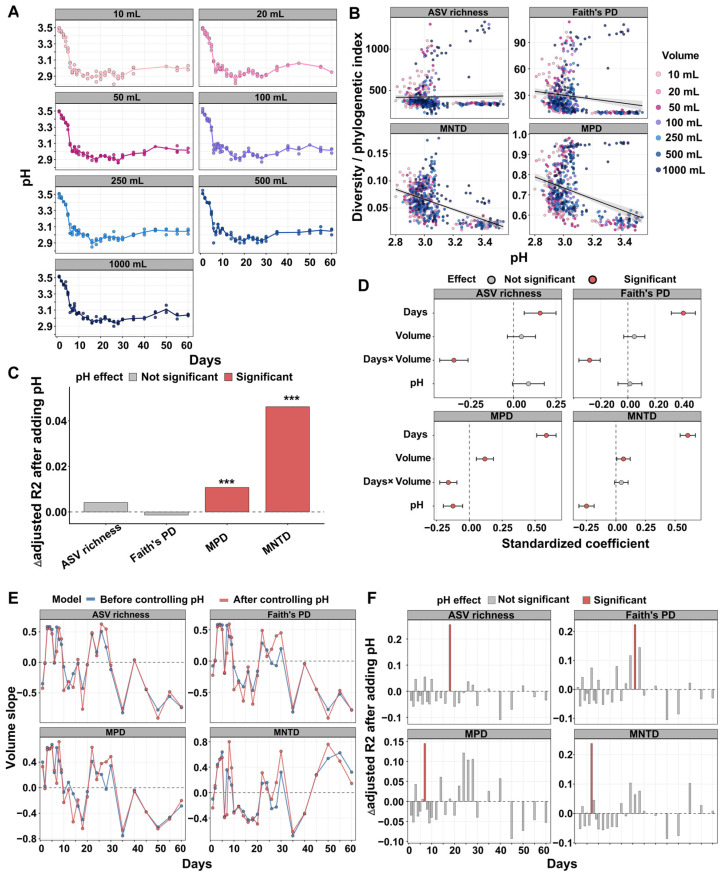
pH dynamics and their explanatory role in diversity and phylogenetic structure. (**A**) pH changes with culture time in the 10, 20, 50, 100, 250, 500, and 1000 mL microcosms. Points represent replicate samples, and lines show mean trends at corresponding time points. (**B**) Relationships between pH and ASV richness, Faith’s PD, MNTD, and MPD. Colors indicate volume treatments, and black lines with gray bands indicate overall regression trends and 95% confidence intervals. (**C**) Changes in adjusted R^2^ after adding pH to the models (*** *p* < 0.001). (**D**) Standardized regression coefficients for Days, Volume, Days × Volume, and pH effects on diversity metrics. Points and error bars indicate coefficient estimates and 95% confidence intervals; dashed vertical lines indicate zero effect. Red indicates significant effects, and gray indicates non-significant effects. (**E**) Changes in daily volume-effect slopes before and after controlling for pH. Blue and red lines represent models without and with pH control, respectively, and the horizontal dashed line indicates a zero slope. (**F**) Changes in adjusted R^2^ after adding pH at each time point. Red bars indicate significant pH effects, and gray bars indicate non-significant effects.

pH exhibited clear temporal dynamics in all volume treatments (Figure 4A). After summarizing by volume and time, mean pH ranged from 2.85 to 3.52, with an overall mean of 3.07. Averaged across time, pH decreased from 3.50 on day 1 to 3.27 on day 5 and 3.03 on day 10, reached a relatively low value of 2.92 on day 18, and remained around 3.02 on day 60. pH was significantly negatively correlated with Days (r = −0.467, *p* = 4.80 × 10^−11^), indicating that acidification was highly synchronized with community succession (Figure 4B).

The increase in model explanatory power after adding pH differed among metrics (Figure 4C). Compared with the baseline Days_z × log10(Volume)_z model, adding pH alone changed adjusted R^2^ by 0.004, −0.001, 0.011, and 0.046 for ASV richness, Faith’s PD, MPD, and MNTD, respectively. After further including Days_z:pH_z and log10(Volume)_z:pH_z interactions, adjusted R^2^ values increased to 0.142, 0.244, 0.480, and 0.605, corresponding to increases of 0.022, 0.028, 0.038, and 0.065 relative to the baseline models.

Standardized regression coefficients showed that pH had a stronger explanatory role for phylogenetic structure metrics than for ASV richness and Faith’s PD as simple additive effects (Figure 4D). In the Add_pH model, the pH_z coefficients for MPD and MNTD were significantly negative (MPD: β = −0.125, *p* = 9.52 × 10^−4^; MNTD: β = −0.249, *p* = 1.21 × 10^−13^), whereas the additive effects of pH_z on ASV richness and Faith’s PD were not significant. In the interaction models, Days_z:pH_z or log10(Volume)_z:pH_z terms were significant for Faith’s PD, MPD, and MNTD in many cases, indicating that pH likely influenced phylogenetic structure through interactions with succession time and habitat volume rather than replacing time effects as an independent environmental factor. Comparisons of daily slopes and adjusted R^2^ before and after controlling for pH further indicated that the explanatory contribution of pH to habitat-size effects was time dependent (Figure 4E,F).

### 3.5. ASV Turnover and Beta-Diversity Decomposition Revealed Sources of Community Change

**Figure 5:**
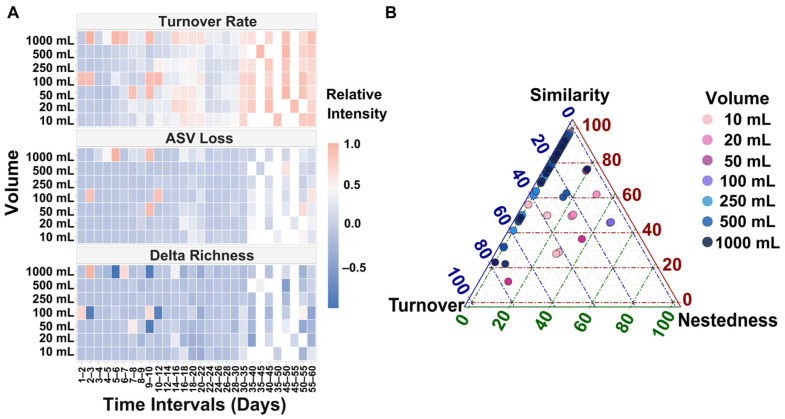
ASV turnover and beta-diversity decomposition between adjacent time intervals. (**A**) Heatmaps of turnover rate, ASV loss, and delta richness across volumes and adjacent time intervals. Colors represent standardized relative intensity, with red indicating higher values and blue indicating lower values. The x-axis represents adjacent sampling intervals, and the y-axis represents volume treatments. (**B**) Ternary decomposition of beta diversity based on the Bray–Curtis framework. The three vertices represent similarity, turnover, and nestedness. Points represent observations from different volumes and time intervals, and colors indicate volume treatments.

ASV turnover between adjacent time intervals showed clear temporal and volume dependence (Figure 5A). Turnover rate ranged from 0.161 to 0.791, with a mean of 0.444; ASV loss ranged from 25 to 1138, with a mean of 201.99; and delta richness ranged from −1079 to 1303, with a mean of 18.81. The highest turnover rate occurred in the 1000 mL treatment between days 2 and 3 (0.791), while the greatest ASV loss occurred in the 1000 mL treatment between days 5 and 6 (1138 ASVs), which also corresponded to the strongest decrease in richness (delta richness = −1079).

Bray–Curtis beta-diversity decomposition showed that adjacent time intervals generally retained high similarity (Figure 5B). Bray–Curtis similarity ranged from 0.121 to 0.973, with a mean of 0.815; Bray–Curtis turnover ranged from 0.027 to 0.769, with a mean of 0.162; and Bray–Curtis nestedness ranged from 0.000 to 0.461, with a mean of 0.022. The highest Bray–Curtis turnover occurred in the 50 mL treatment between days 45 and 50 (0.769), and the highest nestedness occurred in the 100 mL treatment between days 50 and 55 (0.461).

Volume–turnover slope analyses indicated that volume effects were concentrated in a few key time intervals. During days 30–35, ASV loss increased significantly with volume (estimate = 50.61, *p* = 0.014), ASV persistence decreased significantly with volume (estimate = −53.42, *p* = 0.001), delta richness declined significantly with volume (estimate = −264.30, *p* = 0.0049), and loss rate also increased with volume (estimate = 0.095, *p* = 0.0064). In contrast, during days 35–40, ASV loss and loss rate decreased significantly with volume (estimates = −238.35 and −0.142, *p* = 0.012 and 0.014), while delta richness increased significantly with volume (estimate = 278.66, *p* = 0.025). Bray–Curtis similarity also decreased significantly with volume during days 4–5 and 22–24 (estimates = −0.047 and −0.061, *p* = 0.046 and 0.003), suggesting that larger volumes corresponded to stronger compositional differences during certain stages.

### 3.6. Fermentation Time Drove Stage-Dependent Differentiation in Abundance-Weighted Phylogenetic Beta Diversity

**Figure 6:**
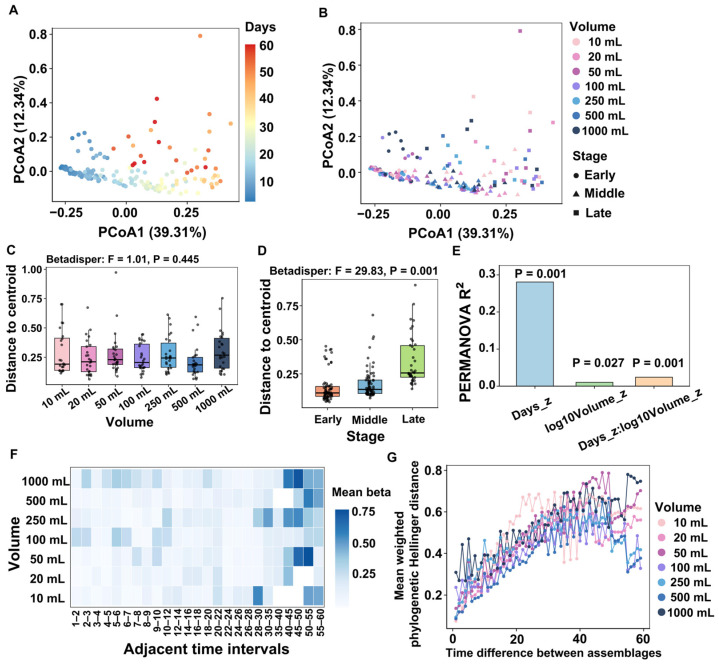
Abundance-weighted phylogenetic beta diversity reveals stage-dependent differentiation in dominant lineage composition. (**A**) PCoA ordination based on abundance-weighted phylogenetic Hellinger distance, with point color indicating sampling day. (**B**) The same PCoA ordination grouped by habitat size and fermentation stage, with color indicating volume treatment and point shape indicating stage. (**C**) Betadisper results among volume treatments; the y-axis represents distances to group centroids. (**D**) Betadisper results among fermentation stages. (**E**) Explanatory contributions of Day, log10(Volume), and their interaction in PERMANOVA. (**F**) Mean abundance-weighted phylogenetic Hellinger distance between adjacent sampling intervals within the same volume treatment; darker colors indicate higher mean phylogenetic beta diversity, and blanks indicate missing valid assemblage pairs. (**G**) Relationship between phylogenetic beta diversity and temporal distance among assemblages within the same volume treatment.

Abundance-weighted phylogenetic beta-diversity analysis showed that the composition of dominant lineages changed markedly during fermentation. PCoA ordination showed that samples separated mainly along the PCoA1 axis according to fermentation time, with PCoA1 and PCoA2 explaining 39.31% and 12.34% of the variation, respectively (Figure 6A,B). PERMANOVA indicated that Day had a significant effect on abundance-weighted phylogenetic beta diversity (*p* = 0.001), that log10(Volume) was also significant (*p* = 0.027), and that the Day × log10(Volume) interaction was significant (*p* = 0.001; Figure 6E), indicating that habitat-size effects changed with fermentation time.

Betadisper analysis showed that within-group phylogenetic dispersion did not differ significantly among volume treatments (F = 1.01, *p* = 0.445; Figure 6C) but differed significantly among fermentation stages (F = 29.83, *p* = 0.001; Figure 6D), with the Late stage showing the highest dispersion. Adjacent-interval and time-lag analyses further showed that later periods and longer time intervals corresponded to higher abundance-weighted phylogenetic Hellinger distances, indicating that differences in dominant lineage composition accumulated as fermentation proceeded (Figure 6F,G).

## 4. Discussion

### 4.1. Habitat-Size Effects Shifted Across Fermentation Succession

This study shows that the relationship between diversity and habitat size in paocai fermentation microcosms is not a stable positive relationship but undergoes a clear stage-dependent transition during community development. In the early stage, both ASV richness and Faith’s PD increased with volume, indicating that larger liquid microcosms maintained more ASVs and longer phylogenetic branches during community establishment. This result is consistent with the basic expectation of the classic SAR, in which larger habitats generally contain more species or taxonomic units [1,2,3]. At the same time, the synchronous increase in Faith’s PD indicates that larger volumes increased not only the number of ASVs but also the amount of evolutionary history represented by phylogenetic branches [7].

However, this early positive effect did not persist throughout the entire fermentation process. During the middle stage, the volume effects on ASV richness and Faith’s PD were markedly weakened, whereas in the late stage they were mostly negative or nonsignificant. Temporal trajectories across volume treatments likewise showed that diversity was generally higher in larger-volume treatments during the early stage, converged among volumes during the middle stage, and was maintained at higher levels in some small- or medium-volume treatments during the late stage. These patterns indicate that, in rapidly successional microbial systems, habitat-size effects cannot be simply interpreted as ‘larger volume supports higher diversity’; instead, they are strongly modulated by the developmental stage of the community. Volume gradients may also influence late-stage reversals through multiple physical and ecological attributes: smaller systems typically have higher surface-area-to-volume ratios, different liquid-to-headspace gas ratios, and faster local resource depletion or metabolic-product accumulation, whereas larger systems may have greater initial population capacity and a higher probability of retaining rare lineages, but may also experience more rapid lineage loss or dominance by prevailing taxa after acidification and the expansion of dominant groups. Together, these processes indicate that the direction of volume effects depends on the stage-specific combination of resources, acidification, gas exchange, and community reassembly.

### 4.2. Asynchrony Between SAR and PDSR Indicates That Taxonomic Richness Cannot Fully Represent Phylogenetic Diversity

ASV richness and Faith’s PD exhibited consistent positive volume responses in the early stage but became asynchronous in the middle and late stages. This pattern indicates that taxonomic richness and phylogenetic diversity, although related, are not interchangeable. ASV richness reflects only the number of detected ASVs, whereas Faith’s PD is also influenced by the positions of ASVs on the phylogenetic tree and by branch lengths. If increasing volume mainly retains closely related ASVs, ASV richness may increase while Faith’s PD increases only slightly. Conversely, if phylogenetically distinctive ASVs are lost, Faith’s PD may decline substantially even when richness changes little [7,8,27].

In this study, SAR and PDSR were largely synchronized in the early stage, suggesting that larger volumes retained both more ASVs and more phylogenetic branches during community establishment. After the middle stage, the two relationships began to decouple: ASV richness could remain positively related to volume at some time points, whereas Faith’s PD had already weakened or become negative. This suggests that the ASVs retained or added during the middle stage may not have contributed a proportional amount of phylogenetic branch length, or that the loss of distantly related low-abundance lineages offset the effect of increased ASV number. PDSR is therefore not a simple extension of SAR but an independent dimension that reveals how communities retain evolutionary history.

### 4.3. Rare ASVs Dominated the Pattern, Whereas the Possible Resurgence of Dormant Lineages in Small Volumes Contributed to Pattern Reversal

The ASV abundance-class structure showed that rare ASVs were the primary source of total ASV richness, consistent with the recognized importance of the microbial rare biosphere in community diversity and temporal dynamics [29,39,40]. In the early stage, rare ASV numbers were highest in larger-volume treatments, indicating that the early positive SAR was largely derived from low-abundance taxa. Because rare ASVs accounted for a very high proportion of total richness, and because some of them were distributed across multiple phylogenetic branches, they likely also contributed to Faith’s PD. Thus, the synchronous increase in ASV richness and Faith’s PD in larger volumes during the early stage may be related to the retention of more low-abundance ASVs and lineage branches.

The negative or non-significant SAR and PDSR observed in the late stage may be related to differences among volume treatments in the recovery of low-abundance or potentially dormant taxa. Microbial dormancy can form a “seed bank”, in which some microorganisms persist in low-metabolic or low-abundance states under unfavorable conditions and become active or detectable again when conditions change [41,42]. In this study, ASV richness and Faith’s PD increased in some small or intermediate volumes in the late stage, whereas such recovery was less evident in large volumes. A plausible explanation is that some ASVs that had previously remained at low abundance, below detection, or in a potentially dormant state reappeared or increased in relative abundance in small microcosms during the late stage, thereby increasing ASV richness and Faith’s PD. In contrast, large volumes may have maintained stronger dominance by abundant taxa or stronger environmental filtering, limiting the recovery of low-abundance taxa.

It should be emphasized that this study was based on 16S rRNA amplicon data and did not directly measure microbial dormancy, metabolic activity, or resuscitation. Therefore, “resurgence of potentially dormant taxa” should be regarded as a possible mechanism explaining the late-stage pattern shift rather than a directly demonstrated physiological process. Nevertheless, this interpretation is consistent with microbial seed-bank theory and helps explain why the early advantage of large volumes weakened and even shifted toward higher diversity in small or intermediate volumes. The late negative relationship therefore does not necessarily mean that small volumes are always more favorable for maintaining diversity; rather, it may reflect a stage-dependent outcome of low-abundance taxon recovery, dominant-taxon replacement, and environmental filtering.

### 4.4. Dominant-Taxon Replacement Altered the Phylogenetic Diversity–Volume Relationship

The relative-abundance composition of major ASVs showed clear replacement of dominant taxa during fermentation. Early communities contained more Proteobacteria-related taxa and low-abundance ASVs, whereas *Lactobacillus*, *Lactococcus*, *Leuconostoc*, and other fermentation-associated lactic acid bacteria became increasingly dominant as fermentation progressed. This transition from multi-taxon coexistence to enhanced dominance of lactic acid bacteria is a common direction of succession in paocai and kimchi fermentation systems and is consistent with declining pH and acidification [43,44].

Dominant-taxon replacement can partly explain the weakening or reversal of PDSR in the middle and late stages. In the early stage, larger volumes retained more ASVs and phylogenetic branches, resulting in higher ASV richness and Faith’s PD. In the middle and late stages, as fermentation-associated dominant taxa increased, community composition became concentrated in fewer dominant lineages. If newly detected or maintained ASVs were mainly concentrated within closely related fermentation-associated groups, ASV richness could remain at a certain level while Faith’s PD increased only slightly. At the same time, the loss of low-abundance taxa or Proteobacteria-related lineages that were more dispersed phylogenetically could further reduce Faith’s PD.

Therefore, stage-dependent PDSR variation was not determined solely by ASV number, but by the combined effects of low-abundance lineage retention, dominant-taxon replacement, and the distribution of ASVs across phylogenetic branches. Early positive PDSR primarily reflected retention of multi-lineage rare taxa in larger volumes, whereas late weakening or reversal of PDSR reflected changes in phylogenetic branch coverage after community reorganization toward dominant fermentation lineages. This also explains why ASV richness and Faith’s PD were asynchronous at certain time points.

### 4.5. pH Changes Mainly Participated in Phylogenetic Structure Adjustment but Did Not Fully Explain Volume Effects

pH declined continuously during fermentation and was significantly correlated with fermentation time, indicating that acidification was one of the most important environmental changes in this system. It should be noted that pH was highly coupled with time, community composition, and microbial metabolic processes. Thus, pH may represent an outcome of metabolic activity by lactic acid bacteria and other taxa, may further promote the dominance of acid-tolerant lineages through environmental filtering, and may also act as a mediating variable linking fermentation stage, volume differences, and community structure. After pH was added to the models, explanatory power increased differently among metrics, with stronger effects on MPD and MNTD than on ASV richness and Faith’s PD as simple additive terms. MPD and MNTD reflect deeper and shallower phylogenetic structure, respectively, and their higher sensitivity to pH suggests that acidification more strongly altered the distribution of relatedness within communities than the number of ASVs alone. This result is consistent with a central idea of phylogenetic community ecology: when environmental filtering is strong and relevant ecological traits are phylogenetically conserved, communities may show phylogenetic clustering or altered phylogenetic structure [8,16]. In this study, fermentation acidification may have filtered for taxa better adapted to low pH, allowing acid-tolerant or fermentation-associated lineages to dominate. The temporal changes in MPD and MNTD and their relatively strong relationships with pH indicate that acidification participated in the reorganization of phylogenetic structure.

pH did not fully replace the effects of time and volume. Even after including pH, time, volume, and their interaction retained explanatory power for some metrics. This indicates that community changes during fermentation cannot be explained by acidification alone but instead resulted from the combined effects of time, volume differences, pH change, dominant-taxon replacement, and ASV turnover. In particular, interactions between pH and time or volume increased the explanatory power for some phylogenetic metrics, suggesting that acidification likely modified the way community phylogenetic structure responded to stage and volume rather than directly determining the direction of SAR or PDSR as a single driver.

### 4.6. ASV Turnover and Phylogenetic Beta Diversity Revealed Enhanced Late-Stage Community Differentiation

ASV turnover analysis showed that community changes between adjacent time intervals were both time and volume dependent. Overall, adjacent intervals maintained high Bray–Curtis similarity, suggesting that community composition did not change abruptly at every stage. However, ASV loss, delta richness, and turnover rate increased markedly during several key time windows, indicating rapid community reorganization at specific stages. Beta-diversity decomposition can further distinguish species replacement, abundance-gradient components, and nested loss, thereby helping determine whether community differences arise primarily from taxon replacement or taxon loss [33,34]. In this study, the strong volume effects on ASV loss and delta richness during some late intervals indicated that late-stage SAR and PDSR changes were closely linked to ASV loss, recovery, and compositional reorganization.

Abundance-weighted phylogenetic beta diversity further showed that fermentation time was the main driver of dominant lineage composition. In the PCoA ordination, samples were distributed along the time gradient, and PERMANOVA indicated that Day had the strongest effect on abundance-weighted phylogenetic beta diversity, while log10(Volume) and the Day × log10(Volume) interaction were also significant. This demonstrates that volume effects were not constant but changed with fermentation time. Betadisper showed no significant difference in phylogenetic dispersion among volume treatments, but dispersion differed significantly among fermentation stages and was highest in the Late stage. Thus, late-stage community differences mainly reflected stage-dependent differentiation in dominant lineage composition rather than simple grouping by volume alone.

Phylogenetic beta-diversity analysis provides complementary evidence for explaining PDSR dynamics. Faith’s PD describes the total phylogenetic branch length retained within a single assemblage, whereas abundance-weighted phylogenetic beta diversity reveals differences in dominant lineage composition among assemblages. Increased dispersion of phylogenetic beta diversity in the Late stage indicates that the dominant lineage structures of communities became more differentiated across time points and volumes. This differentiation helps explain why late-stage SAR and PDSR no longer maintained the early positive relationship: as communities developed toward different dominant lineage structures, the effect of volume on diversity was increasingly modulated by stage-dependent community reorganization.

### 4.7. Implications for Microbial Habitat-Size Effect Studies

The central implication of this study is that microbial habitat-size effects must be understood in the temporal context of community development. Larger habitats do not necessarily maintain higher diversity at all stages. In microbial systems undergoing rapid environmental change, habitat-size effects can be shaped jointly by acidification, resource consumption, dominant-taxon replacement, recovery of low-abundance taxa, and community turnover. Therefore, predictions or management of microbial diversity should consider not only habitat size itself but also community stage, environmental conditions, and rare-lineage dynamics. Integrating SAR, PDSR, phylogenetic structure, and beta diversity can provide a more complete understanding of diversity changes in closed, acidifying, and resource-limited microbial habitats.

Future studies could extend the microbial PDSR framework in three directions. First, PDSR dynamics should be compared between open and closed systems to distinguish the relative contributions of continuous dispersal input, local extinction, and ecological drift to phylogenetic diversity. Previous microbial SAR work has highlighted the importance of resource limitation [45]. Second, the 16S rRNA gene sequencing used in this study cannot distinguish active cells from inactive (dormant) cells; future studies could employ RNA-based sequencing approaches, such as metatranscriptomic sequencing, to better test this hypothesis. Phylogenetic diversity should be integrated with functional traits, genomic potential, and metabolic functions, and attention should be paid to the possibility that sequencing-data processing algorithms and model-fitting procedures may influence the interpretation of diversity–area relationships [46]. Third, similar time dependence should be tested in more complex microbial ecosystems, including soil aggregates, aquatic particles, plant rhizospheres, and animal guts. However, these systems involve open dispersal, spatial heterogeneity, host filtering, and/or multitrophic interactions, and therefore cannot be directly inferred from the closed paocai fermentation system examined here. Watershed-scale ecological perspectives further suggest that microbial geographic patterns may be regulated by larger spatial units and historical processes [47]. From a broader ecosystem-management perspective, PDSR could also be linked with integrative concepts such as holdiversity to connect evolutionary-history retention, diversity maintenance, and sustainability goals [48]. Through such comparisons, PDSR may develop from an empirical description of phylogenetic diversity across habitat scales into a broader theoretical framework for explaining microbial community assembly, evolutionary-history retention, and ecological-function maintenance.

## 5. Conclusions

Using closed paocai fermentation microcosms as a model, this study placed the phylogenetic diversity–area relationship (PDSR) within a continuous successional time series and found that PDSR was not a stable scaling relationship but shifted markedly in direction across fermentation stages. Larger volumes retained more ASVs and phylogenetic branches in the early stage, producing positive SAR/PDSR patterns; in the middle stage, volume effects weakened and the responses of taxonomic richness and Faith’s PD began to decouple; and in the late stage, diversity increased in some small or intermediate volumes, causing PDSR to become negative or non-significant.

Rare ASV retention, replacement by dominant lactic acid bacteria, and acidification jointly formed the main mechanisms underlying PDSR temporal dynamics. Rare ASVs constituted the majority of ASV richness, and the early advantage of large volumes mainly reflected retention of more low-abundance lineages. As fermentation progressed, declining pH and the increasing dominance of lactic acid bacteria reshaped phylogenetic structure, including MPD and MNTD, and weakened or altered the effect of volume on Faith’s PD. ASV turnover and abundance-weighted phylogenetic beta diversity further showed that fermentation time drove stage-dependent differentiation in dominant lineages, while volume effects exhibited non-stationary characteristics through interactions with time.

Accordingly, microbial PDSR should be understood as a dynamic relationship regulated by successional stage rather than as a fixed response to habitat size. By extending the SAR/PDSR framework into the temporal dimension, this study emphasizes that taxonomic diversity, phylogenetic diversity, rare-taxon dynamics, and beta-diversity differentiation must be integrated to explain the formation and transformation of microbial diversity spatial patterns in closed, acidifying, and resource-limited systems.

## Data Availability

The original data presented in the study are openly available in the Genome Sequence Archive (GSA) of National Genomics Data Center, China National Center for Bioinformation/Beijing Institute of Genomics, Chinese Academy of Sciences at https://bigd.big.ac.cn/gsa/browse/CRA008411 (accession number: CRA008411, accessed on 10 May 2026).

## Abbreviations

The following abbreviations are used in this manuscript:

Abbreviation	Definition
ASV	Amplicon sequence variant
FDR	False discovery rate
MNTD	Mean nearest taxon distance
MPD	Mean pairwise distance
PCoA	Principal coordinates analysis
PD	Phylogenetic diversity
PDAR	Phylogenetic diversity–area relationship
PDSR	Phylogenetic diversity–habitat size relationship
PERMANOVA	Permutational multivariate analysis of variance
SAR	Species–area relationship
